# Development and diagnostic validation of a one-step multiplex RT-PCR assay as a rapid method to detect and identify Nervous Necrosis Virus (NNV) and its variants circulating in the Mediterranean

**DOI:** 10.1371/journal.pone.0273802

**Published:** 2022-08-26

**Authors:** Francesca Errani, Enrico Volpe, Enrique Riera-Ferrer, Monica Caffara, Francesc Padrós, Andrea Gustinelli, Marialetizia Fioravanti, Sara Ciulli

**Affiliations:** 1 Department of Veterinary Medical Sciences, Alma Mater Studiorum, University of Bologna, Cesenatico (FC), Italy; 2 Facultat de Veterinària, Universitat Autònoma de Barcelona, Barcelona, Spain; 3 Department of Veterinary Medical Sciences, Alma Mater Studiorum, University of Bologna, Ozzano dell’Emilia (BO), Italy; Taif University, SAUDI ARABIA

## Abstract

Nervous Necrosis Virus (NNV) represents one of the most threatening pathogens for Mediterranean aquaculture. Several NNV strains are currently co-circulating in the Mediterranean Basin with a high prevalence of the RGNNV genotype and the RGNNV/SJNNV reassortant strain and a more limited diffusion of the SJNNV genotype and the SJNNV/RGNNV reassortant. In the present study, a one-step multiplex RT-PCR (mRT-PCR) assay was developed as an easy, cost-effective and rapid diagnostic technique to detect RGNNV and the reassortant RGNNV/SJNNV strain and to distinguish them from SJNNV and the reassortant SJNNV/RGNNV strain in a single RT-PCR reaction. A unique amplification profile was obtained for each genotype/reassortant enabling their rapid identification from cell culture lysates or directly from brain tissues of suspected fish. The method’s detection limit varied between 10^2.3^ and 10^3.4^ TCID_50_ ml^-1^ depending on viral strains. No cross-reacitivty with viruses and bacteria frequently associated with gilthead seabream, European seabass and marine environment was observed. The mRT-PCR was shown to be an accurate, rapid and affordable method to support traditional diagnostic techniques in the diagnosis of VNN, being able to reduce considerably the time to identify the viral genotype or the involvement of reassortant strains.

## Introduction

Nervous necrosis virus (NNV), within the *Betanodavirus* genus, *Nodaviridae* family [1], is the aetiological agent for viral nervous necrosis (VNN), otherwise known as viral encephalopathy and retinopathy (VER), a neuropathological condition reported in at least 62 marine species [2].

Betanodaviruses are small, non-enveloped, single-stranded positive-sense RNA viruses, and their genome consists of two segments named RNA1 (3.1 kb) and RNA2 (1.4 kb), which encode for the RNA-dependent RNA polymerase (RdRp) and the coat protein, respectively [1]. Betanodaviruses are currently classified into four different genotypes officially recognised by the ICTV—International Committee on Taxonomy of Viruses: *Striped jack nervous necrosis virus* (SJNNV), *Redspotted grouper nervous necrosis virus* (RGNNV), *Barfin flounder nervous necrosis virus* (BFNNV) and *Tiger puffer nervous necrosis virus* (TPNNV) [1]. At first, genotyping was based on the analysis of RNA2; however later studies confirmed the same clustering for RNA1 analysis [3–5]. Furthermore, reassortant strains have emerged from the reassortment between the RGNNV and the SJNNV genotypes and named RGNNV/SJNNV (containing the RNA1 deriving from the RGNNV-type and the RNA2 originating from the SJNNV-type) and SJNNV/RGNNV (containing the RNA1 deriving from SJNNV-type and the RNA2 originating from RGNNV-type) [5,6].

Nervous necrosis virus infections are causing severe problems, mainly in the Mediterranean, Asian and Australian marine aquaculture industry [2].

Historically, the RGNNV strain was considered the most important viral pathogen affecting Mediterranean aquaculture, causing severe mortality outbreaks in European seabass (*Dicentrarchus labrax*) and being widely spread in both farmed and wild aquatic animals [7–10]. However, the unpredictable and tremendous ability to evolve of betanodaviruses has led to the co-circulation of different NNV strains in the Mediterranean Basin. Currently, RGNNV genotype and the reassortant RGNNV/SJNNV are the dominant strains infecting both gilthead seabream (*Sparus aurata*) and European seabass, posing a severe threat to the Mediterranean marine finfish industry [11–13]. The RGNNV/SJNNV reassortant strain is widespread in the Mediterranean and has been isolated from farmed European sea bass, common sole (*Solea solea*), Senegalese sole (*Solea senegalensis*) and gilthead sea bream. In contrast, SJNNV/RGNNV reassortants have only been isolated from European sea bass in Italy and Croatia [5,6,11,14], while SJNNV was detected in fish reared in Spain and Portugal [15–17].

Several diagnostic techniques are available for NNV detection i.e. electron microscopy, cell culture isolation, immunodetection and molecular methods. However, according to the World Organization for Animal Health (OIE), the ‘Gold Standard’ method to detect NNV is the isolation of viral agents in cell culture, followed by immunological or molecular identification [18].

Furthermore, given the wide co-circulation of different betanodaviruses in the same geographic region and considering their different features in pathogenicity and host and temperature tropism, laboratories are expected not only to detect, but also to genotype isolated or detected viruses [19,20]. However, proficiency tests performed by the OIE reference laboratory pointed out a limited capability to properly identify the NNV variants [19]. In particular, in the 2^nd^ VER interlaboratory proficiency test (VER-IPT) less than half of the participants completed the viral identification and only 2 out of 29 laboratories correctly and completely identified all the viruses included in the panel test [19]. In the 3^rd^ VER-IPT an increasing number of participants completed the viral identification, although their number was still too low to be considered satisfactory [19]. The low capacity to identify the viral genome is partially due to the lack of a easy and fast method widely implementable also in non-specialised laboratories. So far, identification of NNV variants is based on amplification, sequencing and phylogenetic analysis of both viral genome RNA molecules. At present, some approaches regarding PCR-based and blot reactions have been developed in order to discriminate between RGNNV and SJNNV genotypes, reflecting the need for an easy and affordable diagnostic tool to identify the different NNV variants circulating in the Mediterranean. However, these assays remain limited to independent detection of RNA1 and RNA2 viral genome segments, rendering them unable to distinguish parental RGNNV and SJNNV strains from reassortant RGNNV/SJNNV and SJNNV/RGNNV strains in a single reaction [21–28].

As viral identification is an essential information for the management of the disease in the field [19], the development of an easy and affordable method to genotype betanodaviruses was included upon request of the Mediterranean Marine Fish Farming sector in the H2020 Peformfish Project, an industry-driven project targeting industry defined priorities.

In recent years, multiplex PCR assays have gained popularity due to their convenience in terms of cost and time and the ability to simultaneously detect several pathogens or genotyping them [29,30]. In particular, both conventional and real time PCR have successfully been multiplexed to discriminate genetic variants of fish pathogens [29–31].

In general terms, real time PCR shows higher sensitivity than conventional PCR, furthermore it can reduce cross-contamination associated to post-amplification steps and may provide quantitative information [32]. However, real time PCR requests a higher investment in terms of cost and expertise compared to conventional PCR. On the other hand, conventional PCR, despite less performing, can be more affordable on a large scale to diagnostic laboratories including small laboratories supporting aquaculture industry.

The aim of this study was to develop and validate a one-step multiplex conventional RT-PCR assay as an easy and affordable diagnostic technique to support Mediterranean fish production detecting NNV and simultaneously discriminating between RGNNV, SJNNV genotypes, and RGNNV/SJNNV and SJNNV/RGNNV reassortant strains. To achieve this, both RNA1 and RNA2 from RGNNV and RNA2 from SJNNV were targeted in a single RT-PCR reaction.

## Materials and methods

### Multiplex RT-PCR primer design

In order to design primer candidates, two alignments were generated, one for RNA1 and another one for RNA2 viral genome segments. Both alignments included sequences from RGNNV and SJNNV isolates and the reassortant strains (RGNNV/SJNNV and SJNNV/RGNNV) (S1 and S2 Tables). Sequences were obtained from the GenBank database (https://www.ncbi.nlm.nih.gov/genbank/) and aligned with Clustal W implemented in the BioEdit software [33].

RGNNV-RNA1 and RGNNV-RNA2 primers were designed based on conserved regions within each genotype’s sequence and diverging from SJNNV-RNA1 and SJNNV-RNA2, respectively. In addition, SJNNV-RNA1 and SJNNV-RNA2 primers were designed based on conserved regions within each genotype’s sequence and diverging from RGNNV-RNA1 and RGNNV-RNA2, respectively.

Several primers targeting RGNNV-RNA1, RGNNV-RNA2, SJNNV-RNA1 and SJNNV-RNA2 were designed and combined together and with other several primers already described in literature [5,34].

Primer sets were selected according to their position within each genotype’s RNA sequence, the melting temperature (Tm) and the predicted amplicon size to amplify fragments of different sizes specific for RGNNV-RNA1, RGNNV-RNA2 and SJNNV-RNA2.

Newly designed primers were first checked for specificity using NCBI-Nucleotide blast (https://blast.ncbi.nlm.nih.gov/Blast.cgi) as well as for self-dimers and primer dimers via Thermo Fisher Scientific® Multiple Primer Analyzer online software (https://www.thermofisher.com/) and then tested with NNV reference strains representing the RGNNV and SJNNV genotypes and the reassortant strains (RGNNV/SJNNV and SJNNV/RGNNV).

### Viruses

The optimisation of the multiplex PCR assay was carried out using already characterised NNV strains: It/351/Sb (RGNNV) isolated from *D*. *labrax* [35]; 416-Dec17 (RGNNV/SJNNV) isolated from *S*. *aurata* larvae [11]; 389/I96 (SJNNV/RGNNV) isolated from *D*. *labrax* and 484.2.2009 (SJNNV) isolated from *S*. *senegalensis* [14,36].

Viruses were cultured on SSN-1 cell monolayers using 25cm^2^ culture flasks [37]. Infected monolayers were incubated at 20 ± 1°C (SJNNV and SJNNV/RGNNV) and 25 ± 1°C (RGNNV and RGNVV/SJNNV) until cytopathic effects were observed.

RNA from each NNV strain was extracted from cell lysates using NucleoSpin^®^ RNA (Macherey-Nagel GmbH & Co., Germany) according to manufacturer’s instructions and stored at -80°C until use.

### Optimisation of the multiplex RT-PCR assay

First, the individual amplification assays (singleplex) to detect each of the three viral targets (RGNNV-RNA1, RGNNV-RNA2 and SJNNV-RNA2) were conducted under various conditions of primer combination and concentration, and with various annealing temperatures, in order to choose the most suitable primer pair to each target. Then, the PCR primers were multiplexed and PCR conditions, such as primer concentrations, annealing temperature and annealing time, were optimised to amplify only specific targets for each strain. The reaction was tested first with a two step approach using the High Capacity cDNA Reverse Transcription Kit (Applied Biosystem, US) for cDNA synthesis and the Taq DNA Polymerase Recombinant (Invitrogen, USA) for target amplification and finally with a one-step approach using the SuperScript™ III One-Step RT-PCR System with Platinum™ Taq DNA polymerase (Invitrogen, USA). Positive and negative controls were run along with all reactions. Finally, a set of three optimal primer pairs was selected to conduct the one-step multiplex RT-PCR assay and further validation (Table 1).

**Table 1:** Selected primer pairs for the multiplex one-step RT-PCR assay.

Primer name	Sequences (5’→3’)	Position^1^	Target	Amplicon (bp)	Reference
RG_Fw1_RNA1	GACTCAGATCCAGCGGGAA	246–264^a^	RGNNV-RNA1	169	This study
RG_Rev6_RNA1	TCCAACCTCACGGGGTGAT	396–414^a^			
RG_SPCF1b_RNA2	CAATCGTCGGCGTAGTAATC	107–126^a^	RGNNV-RNA2	647	This study
RG_SPCFRev3b_RNA2	AGGAGGATGGACTTGAAGTC	735–754^a^			
SJ_F2_RNA2	ATTACTACCCAGGCGCCAC	691–709^b^	SJNNV-RNA2	350	This study
R3	CGAGTCAACACGGGTGAAGA	1022–1041^b^			[30]

^a^ The position of primers is based on the sequences of strain SGWak97 (GenBank accession numbers for RNA1 AY324869 and RNA2 AY324870).

^b^ The position of primers is based on the sequences of strain SJNNV (GenBank accession number for RNA2 AB056572).

### Analytical specificity of multiplex RT-PCR

The analytical specificity of the multiplex RT-PCR was tested using several viruses and bacteria frequently associated with gilthead seabream, European seabass and marine environment. The assay was performed using total DNA extracted from gilthead sea bream affected by lymphocystis disease virus (LCDV) [38] and DNA extracted from bacteria known to cause disease in gilthead sea bream and European sea bass or associated with the aquatic environment (*Vibrio anguillarum*, *V*. *harveyi*, *V*. *alginolyticus*, *V*. *gigantis*, *Photobacterium damselae* subsp. *piscicida* and *Aeromonas veronii*) [39,40]. Furthermore, the assay was performed using RNA extracted from viral isolates of viral hemorrhagic septicemia virus (VHSV), BFNNV and TPNNV and total RNA extracted from uninfected snakehead-fish cell line (SSN-1) [41].

### Analytical sensitivity of multiplex RT-PCR

In order to determine the limit of detection of the developed one-step multiplex RT-PCR method, the viral titre was calculated for each reference strain. Titrations of viruses were performed according to the end-point dilution method. Briefly, ten-fold serial dilutions (10^−2^ to 10^−9^) of viral solutions were inoculated onto 24h-old SSN-1 cell culture in 96-well plates and incubated at 20 ± 1°C (SJNNV and SJNNV/RGNNV) and 25 ± 1°C (RGNNV and RGNNV/SJNNV). The viral adsorption period was 1h for each strain. Daily readings were carried out up to one-week post-inoculation. The titre was expressed as TCID_50_ ml^-1^ and calculated according to the Spearman-Karber method [42].

Limits of detection for the one-step multiplex RT-PCR were established individually for each reference strain using extracted RNA from at least five viral dilutions. RNA extracted from dilutions was also subjected to the real-time RT-PCR method validated by the OIE.

### Repeatability (intra- and inter-assay variance)

To test for precision and robustness of the multiplex RT-PCR, RNA extracted from brains (n = 3) was analysed in triplicates in the same assay (intra-assay variance). RNA samples were subjected to both the real-time RT-PCR [25] and the novel mRT-PCR assay. According to the real time RT-PCR these samples were negative (sample 26/19), weakly positive (sample 156/18; Ct-value 29.6), and distinct positive (sample 173/18B; Ct-value 9.9). Furthermore, to test for variation in results between runs (inter-assay variance), the samples used for intra-assay variance were tested as single-preparations on two consecutive days.

### Diagnostic sensitivity and specificity

Seventy-six samples including 50 European seabass and 23 gilthead sea bream brains and 3 *Artemia salina* (nauplii) samples were used to determine diagnostic sensitivity and specificity of the mRT-PCR (S3 Table).

RNA was extracted from brain samples using PureLink^TM^ RNA mini kit (Thermo Fisher Scientific, USA) according to manufacturer’s instructions and stored at -80°C until use.

All samples were subjected to parallel testing using the one-step multiplex RT-PCR method and the real-time RT-PCR protocol validated by the OIE reference centre for viral encephalopathy and retinopathy [25].

The one-step multiplex RT-PCR assay was conducted using the optimised protocol.

The real-time RT-PCR assay was carried out according to Baud and colleagues [24] using the specific primers oPVP154 (5’TCCAAGCCGGTCCTAGTCAA3’), oPVP155 (5’CACGAACGTKCGCATCTCGT 3’) and probe (Cy5-CGATCGATCAGCACCTSGTC-BHQ2). Briefly, the reaction mixture contained 1x of Quantitect RT-PCR master mix (Qiagen), 600 nM of each primer, 400 nM of the probe, 2.5 μl of RNA in 12.5 μl total volume. The thermal cycling conditions were 30 min at 50°C, followed by 15 min at 95°C, and 40 cycles of denaturation/extension for 15 sec at 94°C and 60 sec at 60°C.

mRT-PCR results were compared to the real-time RT-PCR [25] taken as the “Gold Standard”. Specificity, sensitivity, positive predictive value (probability that a test-positive fish is true-positive), and negative predictive value (probability that a test-negative fish is true-negative) of the mRT-PCR were calculated according to Thrusfield and colleagues [43].

### Viral encephalo-retinopathy interlaboratory proficiency test

To further test the robustness of the mRT-PCR, 10 freeze-dried samples (NNV-positive n = 5, NNV-negative n = 5), included in the 3^rd^ VER-IPT organised by the OIE reference laboratory for Viral Encephalopathy and Retinopathy [19], were tested. Positive samples consisted of different betanodavirus species, namely RGNNV (10^7.30^ TCID_50_ ml^-1^), SJNNV (10^9.55^ TCID_50_ ml^-1^), and the reassortant strains RGNNV/SJNNV (10^7.55^ and 10^4.55^ TCID_50_ ml^-1^) and SJNNV/RGNNV (10^8.30^ TCID_50_ ml^-1^), whereas negative samples consisted in either MEM with and without 10% yeast extract or negative rainbow trout (*Oncorhynchus mykiss*) serum (S4 Table) [19]. Samples were resuspended in 0.5 ml of Leibovitz L-15 medium (Biowest, USA) according to the VER-IPT instructions, and RNA was extracted from 100μl using NucleoSpin® RNA (Macherey-Nagel GmbH & Co., Germany) according to manufacturer’s instructions and stored at -80°C until use.

### Genotyping

To assess the accuracy of the genotyping performed via the new developed mRT-PCR a selection of positive samples which reported a range of results in the mRT-PCR assay were subjected to conventional genotyping through amplification and sequencing of the RNA1 (n = 5) and the RNA2 (n = 27) viral genome using previously published protocols [5,8]. Briefly, the amplification step was conducted through a one-step RT-PCR assay with primers S6 (5’-ATGGTACGCAAAGGTGATAAGAAA-3’) and S7 (5’-GTTTTCCGAGTCAACACGGGT-3’) targeting the whole coding sequence of RNA2 viral genome using the SuperScript III One-Step RT-PCR System (Invitrogen, Carlsbad, USA).

The reaction mixture contained 1x Reaction Mix, 0.8 μM for each primer, 0.3 μl Superscript III/Platinum Taq enzyme mix and 3 μl RNA in 15 μl total volume. The thermal cycling conditions were 45°C for 30 min, 95°C for 2 min, followed by 40 amplification cycles of 94°C for 60 sec, 58°C for 60 sec and 72°C for 60 sec. A final extension was performed at 72°C for 7 min. To avoid cross-contaminations, the amplification reactions were all set up with negative controls.

PCR products were purified using the Exosap reagent (Affymetrix, Santa Clara, CA) and then sequenced at Bio-Fab Research srl (Rome, Italy). The sequences obtained were corrected manually, aligned and compared with reference strain sequences available in GenBank using Clustal W implemented in the BioEdit software [33].

The phylogenetic analysis was conducted using the maximum likelihood (ML) method, with the general time-reversible (GTR) nucleotide substitution model [44], available in MEGA X software [45]. One thousand bootstrap replicates were performed to assess the robustness of individual nodes, and only values ≥70% were considered significant.

## Results

### Multiplex RT-PCR optimisation

A one-step multiplex RT-PCR targeting RGNNV-RNA1, RGNNV-RNA2 and SJNNV-RNA2 was developed to detect and simultaneously identify the presence of RGNNV, SJNNV or one of their reassortant strains (RGNNV/SJNNV, SJNNV/RGNNV).

Three primer pairs were selected and multiplexed (Table 1), optimal conditions were selected to obtain 1 to 2 specific bands for each viral strain.

The optimised reaction consisted of a reaction mix containing 3 μl RNA, 7.5 μl of 2x Reaction Mix, 0.3 μl of Enzyme mix, 10 μM of each primer and DNase-free water to reach the final volume of 15 μl. The optimal thermal cycling conditions were 45°C for 30 min, 2 min at 94°C followed by 40 amplification cycles of 30 sec at 95°C, 30 sec at 60°C and 30 sec at 72°C. Post-elongation was performed for 10 min at 72°C.

**Fig 1:**
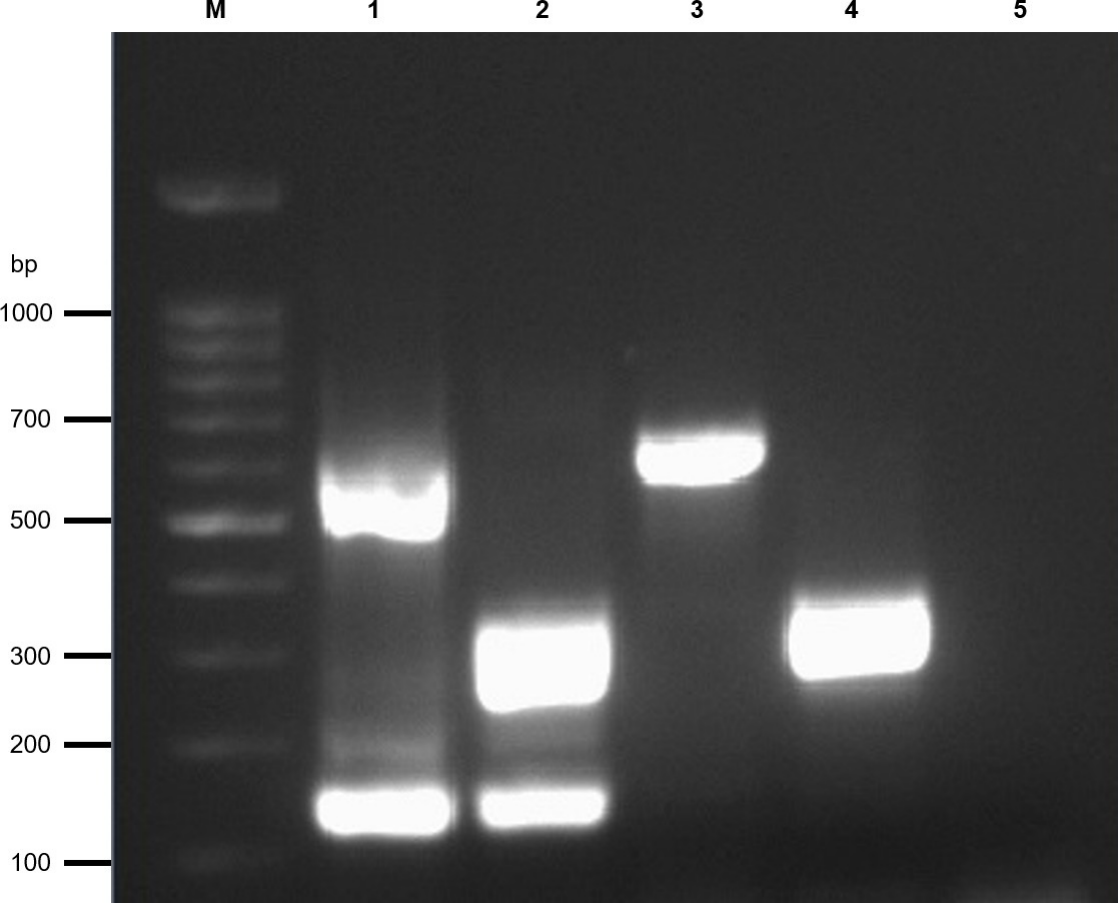
Multiplex RT-PCR. Agarose gel electrophoresis of PCR products amplified from RNA extracted from NNV reference strains. Lane M: 100 bp ladder (Invitrogen, Carlsbad, USA); Lane 1: RGNNV genotype; Lane 2: RGNNV/SJNNV reassortant strain; Lane 3: SJNNV/RGNNV reassortant strain; Lane 4: SJNNV genotype; Lane 5: No template control; Molecular weight markers (bp) are indicated to the left of the gel.

The reference strains used for mRT-PCR optimisation were correctly identified producing the specific bands for each genotype/reassortant strain. The It/351/Sb strain (RGNNV genotype) was identified by two amplification bands one at 169 bp targeting the RGNNV-RNA1 and one at 647 bp targeting the RGNNV-RNA2, the 484.2.2009 strain (SJNNV genotype) was identified by the 350 bp band targeting the SJNNV-RNA2. Regarding the reassortant strains, the 416-Dec17 strain (RGNNV/SJNNV) produced two specific bands one at 169 bp targeting RGNNV-RNA1 and one at 350 bp targeting SJNNV-RNA2 and the 389/l96 strain (SJNNV/RGNNV) generated the 647 bp band targeting the RGNNV-RNA2 (Fig 1).

### Analytical specificity

**Fig 2:**
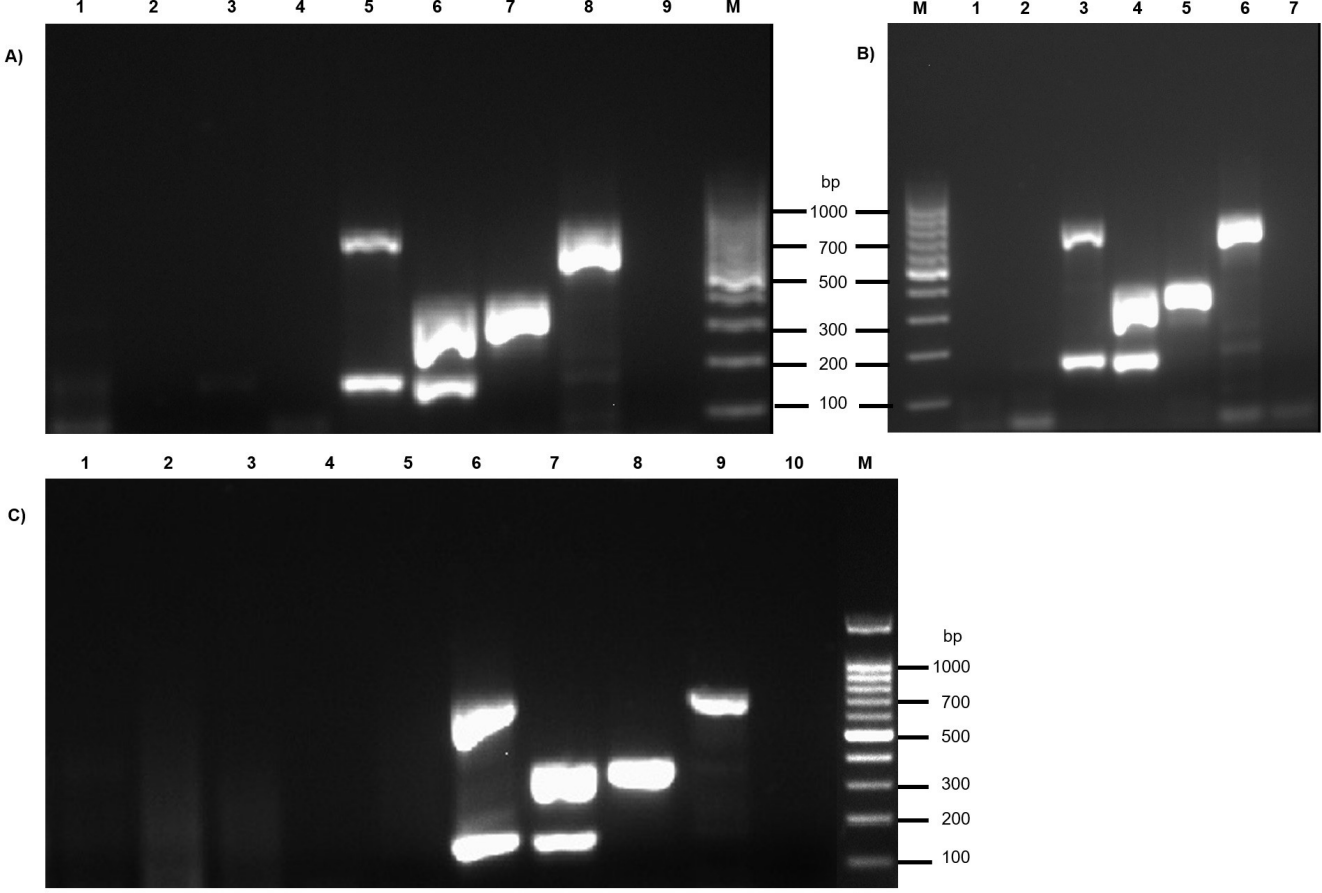
Multiplex RT-PCR specificity test. Agarose gel electrophoresis of PCR products amplified from cell-culture samples and viral and bacterial fish pathogens. A) Lane 1: VHSV; Lane 2: *V*. *harveyi*; Lane 3: *V*. *alginolyticus*; Lane 4: *V*. *gigantis*; Lane 5: RGNNV genotype; Lane 6: RGNNV/SJNNV reassortant strain; Lane 7: SJNNV/RGNNV reassortant strain; Lane 8: SJNNV genotype; Lane 9: No template control; B) Lane 1: BFNNV genotype; Lane 2: TPNNV genotype; Lane 3: RGNNV genotype; Lane 4: RGNNV/SJNNV reassortant strain; Lane 5: SJNNV/RGNNV reassortant strain; Lane 6: SJNNV genotype; Lane 7: No template control; C) Lane 1: SSN1 cell line; Lane 2: LCDV; Lane 3: *V*. *anguillarum*; Lane 4: *P*. *damselae* subsp. *piscicida*; Lane 5: *A*. *veronii*; Lane 6: RGNNV genotype; Lane 7: RGNNV/SJNNV reassortant strain; Lane 8: SJNNV/RGNNV reassortant strain; Lane 9: SJNNV genotype; Lane 10: No template control. Lanes M: 100 bp ladder (Invitrogen, Carlsbad, USA).

No false-positive signals were observed with cell-culture RNA sample nor with any viral and bacterial DNA/RNA (LCDV, VHSV, *V*. *anguillarum*, *V*. *harveyi*, *V*. *alginolyticus*, *V*. *gigantis*, *P*. *damselae* subsp. *piscicida*, *A*. *veronii*). The mRT-PCR assay also excluded the other NNV genotypes (BFNNV and TPNNV). The presence of specific bands in the positive controls has demonstrated a valid test performance. No specific bands were present in the negative control (Fig 2).

### Analytical sensitivity of multiplex RT-PCR

The infectious titres of the produced viral solutions were 10^8.4^, 10^6.8^, 10^7.3^ and 10^5.2^ TCID_50_ ml^-1^ for RGNNV, SJNNV, RGNNV/SJNNV and SJNNV/RGNNV, respectively. The analysis of tenfold dilutions of titrated viral solutions showed a sensitivity ranging from 10^2.3^ to 10^3.4^ TCID_50_ ml^-1^. The highest sensitivity was referred to the detection of the RGNNV/SJNNV strain: the mRT-PCR assay was able to detect the viral RNA extracted from the fifth tenfold dilution of a solution with a titre of 10^7.3^ TCID_50_ ml^-1^ corresponding to an inferred titre of 10^2.3^ TCID_50_ ml^-1^ and a Ct value of 33.7 ± 0.77 at the real time RT-PCR. For RGNNV, SJNNV and SJNNV/RGNNV, the mRT-PCR detected the virus into dilutions with inferred titres of 10^3.4^, 10^2.8^ and 10^3.2^ TCID_50_ ml^-1^, respectively and Ct values of 35.4 ± 0.37, 30.5 ± 0.02 and 24.9 ± 0.11, respectively (Table 2).

**Table 2:** Limit of detection of mRT-PCR determined on tenfold dilution of viral solutions; the titre is expressed as TCID_50_ ml^-1^; the real time RT-PCR results are expressed as Ct values ± standard deviation (SD).

Dilution factor	RGNNV It/351/Sb			SJNNV 484.2.2009			RGNNV/SJNNV Sa-416-Dec17			SJNNV/RGNNV 389/I96		
	mRT-PCR	Virus titre	Real time RT-PCR	mRT-PCR	Virus titre	Real time RT-PCR	mRT-PCR	Virus titre	Real time RT-PCR	mRT-PCR	Virus titre	Real time RT-PCR
**10** ^**−0**^	+	10^8.4^	n.d	+	10^6.8^	n.d	+	10^7.3^	n.d	+	10^5.2^	n.d
**10** ^**−1**^	+	10^7.4^	n.d	+	10^5.8^	+ (18.7 ± 0.17)	+	10^6.3^	n.d	+	10^4.2^	+ (20.7 ± 0.35)
**10** ^**−2**^	+	10^6.4^	+ (25.4 ± 0.04)	+	10^4.8^	+ (23.2 ± 0.28)	+	10^5.3^	+ (23.2 ± 0.11)	+	10^3.2^	+ (24.9 ± 0.11)
**10** ^**−3**^	+	10^5.4^	+ (29.4 ± 0.18)	+	10^3.8^	+ (26.7 ± 0.20)	+	10^4.3^	+ (26.7 ± 0.39)	-	10^2.2^	+ (28.2 ± 0.09)
**10** ^**−4**^	+	10^4.4^	+ (32.2 ± 0.37)	+	10^2.8^	+ (30.5 ± 0.02)	+	10^3.3^	+ (30.0 ± 0.32)	-	10^1.2^	+ (31.5 ± 0.38)
**10** ^**−5**^	+	10^3.4^	+ (35.4 ± 0.37)	-	10^1.8^	+ (33.4 ± 0.02)	+	10^2.3^	+ (33.7 ± 0.77)	n.d	n.d.	n.d.
**10** ^**−6**^	-	10^2.4^	+ (38.0 ± 0.04)	-	10^0.8^	+ (36.0 ± 0.30)	-	10^1.3^	+ (34.8 ± 0.65)	n.d	n.d.	n.d.
**10** ^**−7**^	-	10^1.4^	-	n.d	n.d.	n.d.	-	10^0.3^	-	n.d	n.d.	n.d.
**10** ^**−8**^	-	10^0.4^	-	n.d	n.d.	n.d.	-	10^0.03^	-	n.d	n.d.	n.d.

n.d. not determined.

### Intra and inter-assay variability

**Fig 3:**
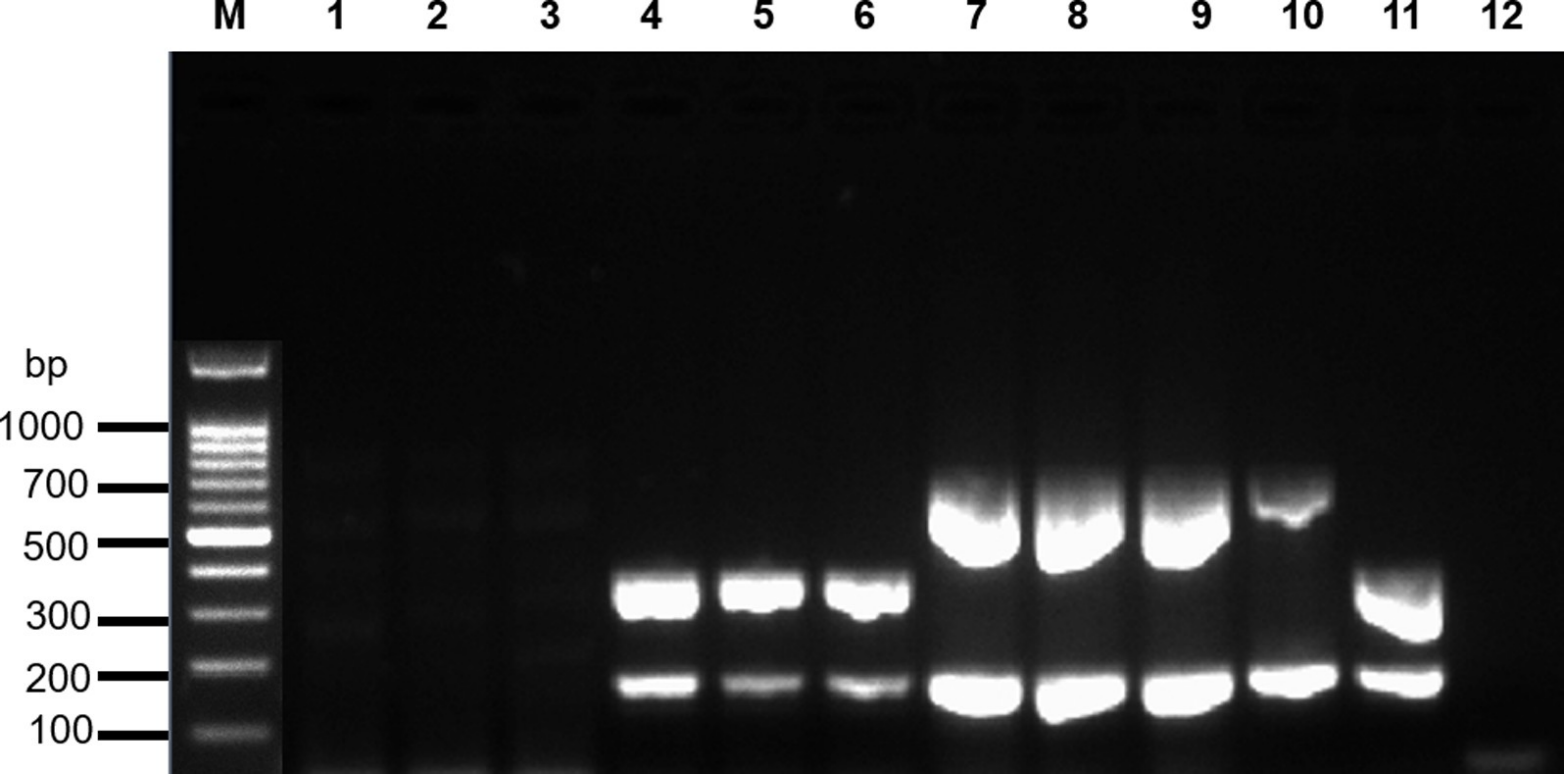
Multiplex RT-PCR repeatability test. Agarose gel electrophoresis of PCR products amplified from RNA extracted from NNV-positive and NNV-negative samples. Lane M: 100 bp ladder (Invitrogen, Carlsbad, USA); Lane 1–3: NNV-negative sample; Lane 4–6: NNV weakly positive sample; Lane 7–9: NNV distinct positive sample; Lane 10: RGNNV genotype; Lane 11: RGNNV/SJNNV reassortant strain; Lane 12: No template control; Molecular weight markers (bp) are indicated to the left of the gel.

No variation in multiplex RT-PCR results for positive and negative samples within and between runs have been observed. All replicates of both weakly (Ct-value 29.6), and distinct positive Ct-value 9.9) samples showed 100% positive results when tested in triplicate within the same multiplex RT-PCR run (intra-assay variability; Fig 3) and when tested in multiplex RT-PCRs at two consecutive days (inter-assay variability). All replicates of negative sample were negative in both intra- and inter-assay variability tests.

### Diagnostic sensitivity and specificity

Diagnostic sensitivity and specificity were tested using RNA from 76 tissue samples extracted from European sea bass, gilthead sea bream and *Artemia salina* (S3 Table). NNV positivity/negativity was established based on the real-time RT-PCR protocol validated by the OIE reference centre for viral encephalopathy and retinopathy [25] regarded as the ‘Gold Standard’ method [43]. According to this protocol, 34 NNV-positive and 42 NNV-negative samples were obtained.

The mRT-PCR accurately detected no NNV-RNA in NNV-negative samples (n = 42), assuming a diagnostic specificity of 100%. Furthermore, all NNV-positive samples (n = 34) produced at least one specific amplification band being identified as positive (diagnostic sensitivity = 100%).

### Viral encephalo-retinopathy interlaboratory proficiency test

**Fig 4:**
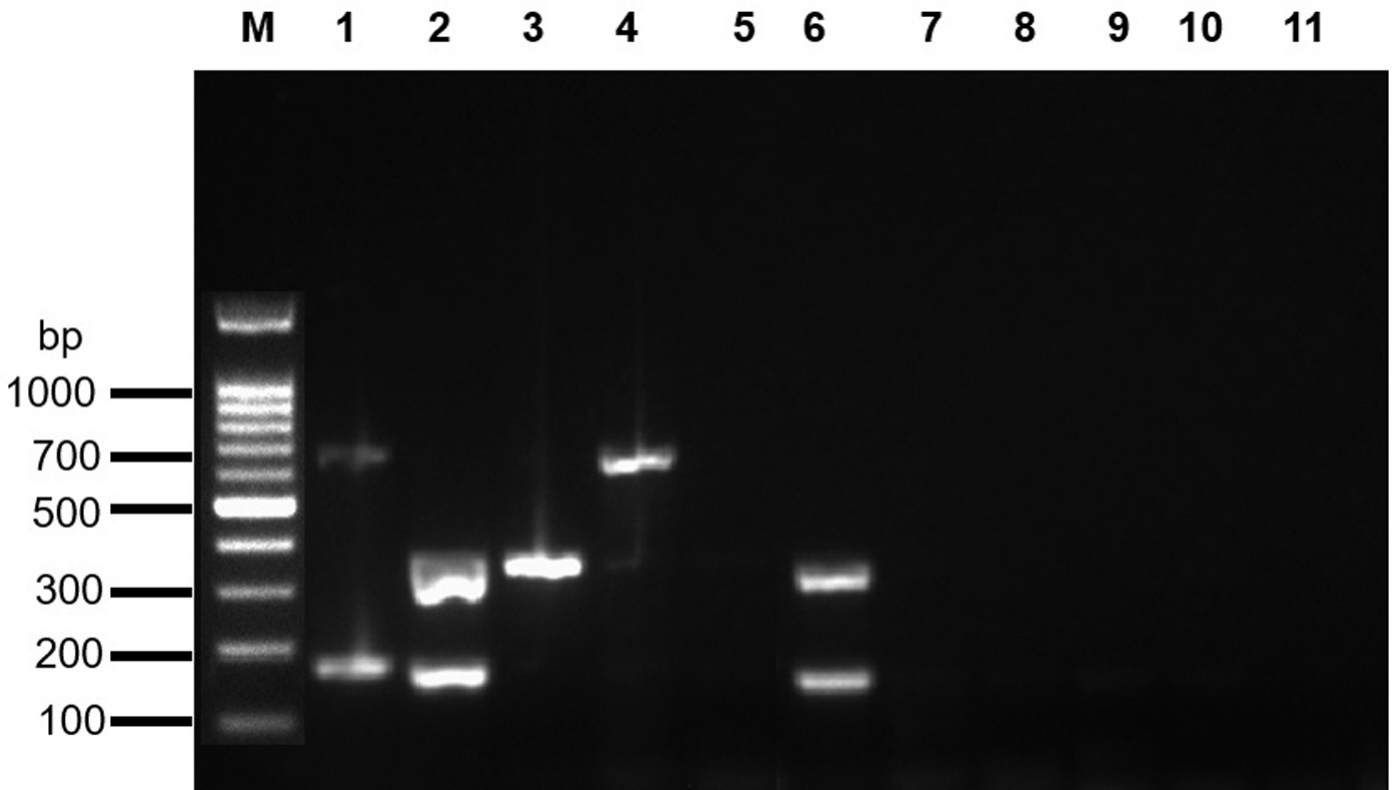
Multiplex RT-PCR applied to VER-IPT samples. Agarose gel electrophoresis of PCR products amplified from RNA extracted from ten VER-IPT samples. Lane M: 100 bp ladder (Invitrogen, Carlsbad, USA); Lane 1–4 and 6: NNV-positive samples; Lane 5 and 7–10: NNV-negative samples; Lane 11: No template control; Molecular weight marker (bp) isindicated to the left of the gel. For sample details see S4 Table.

The application of the mRT-PCR to the samples from the 3^rd^ VER-IPT enabled to correctly detect their positivity/negativity (Fig 4). Furthermore, all positive samples included in the VER-IPT test covering all genotypes (RGNNV and SJNNV) and reassortant strains (RGNNV/SJNNV and SJNNV/RGNNV) were correctly genotyped (Fig 4).

### Genotype identification

Analysis conducted with mRT-PCR allowed us to assign the RNA1 of all the positive analysed samples (34/34) to a genotype (RGNNV-type). In contrast, the RNA2 was genotyped in 30 of the 34 positive samples. Twenty-four samples belonged to RGNNV genotype showing the two specific bands at 169 and 647 bp, whereas 6 samples were RGNNV/SJNNV reassortant strains amplifying two bands of 169 and 350 bp. Four samples showed positivity to the RNA1 amplification band common to RGNNV and RGNNV/SJNNV reassortant strain (169 bp) but did not generate the specific band for RGNNV-RNA2 (647 bp) nor SJNNV-RNA2 (350 bp), not allowing their differentiation.

**Fig 5:**
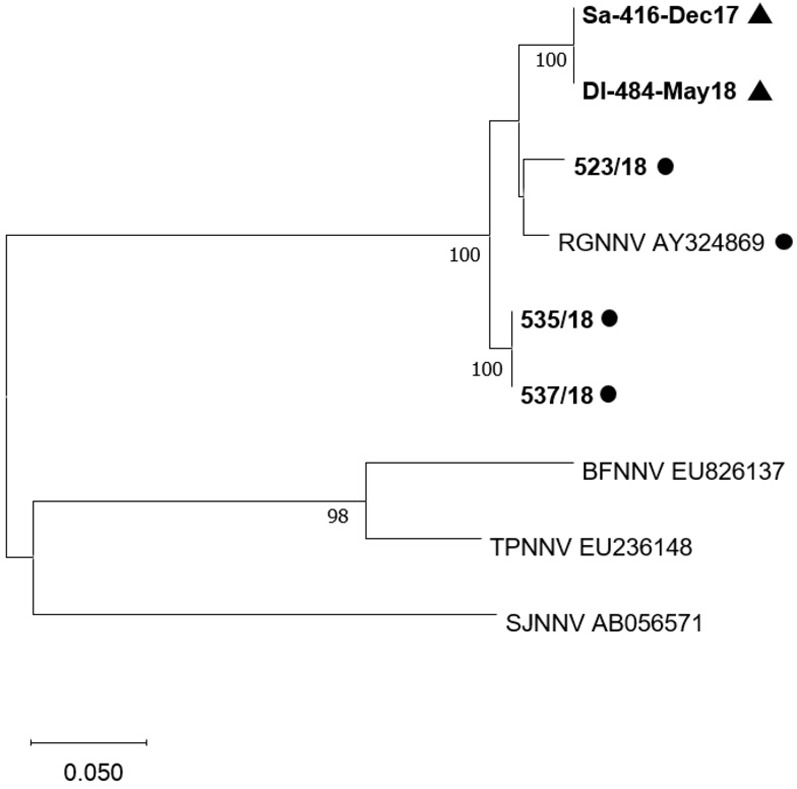
Phylogenetic tree based on RNA1 sequences. Phylogenetic tree based on RNA1 nucleotide sequences. Reference strains are reported with genotype name and accession number. All sequences of tested samples (bold) including parental RGNNV (dot) and RGNNV/SJNNV reassortant strains (triangle) clustered within the RGNNV group. Bootstrap values>70% are shown. Branch lengths are scaled according to the number of nucleotide substitutions per site. The scale bar is reported.

Conventional genotyping through amplification and sequencing of the RNA1 and RNA2 fragments confirmed mRT-PCR genotype identification. All sequenced RNA1 fragments were attributed to RGNNV-type including parental RGNNV and reassortant strains (Fig 5).

**Fig 6:**
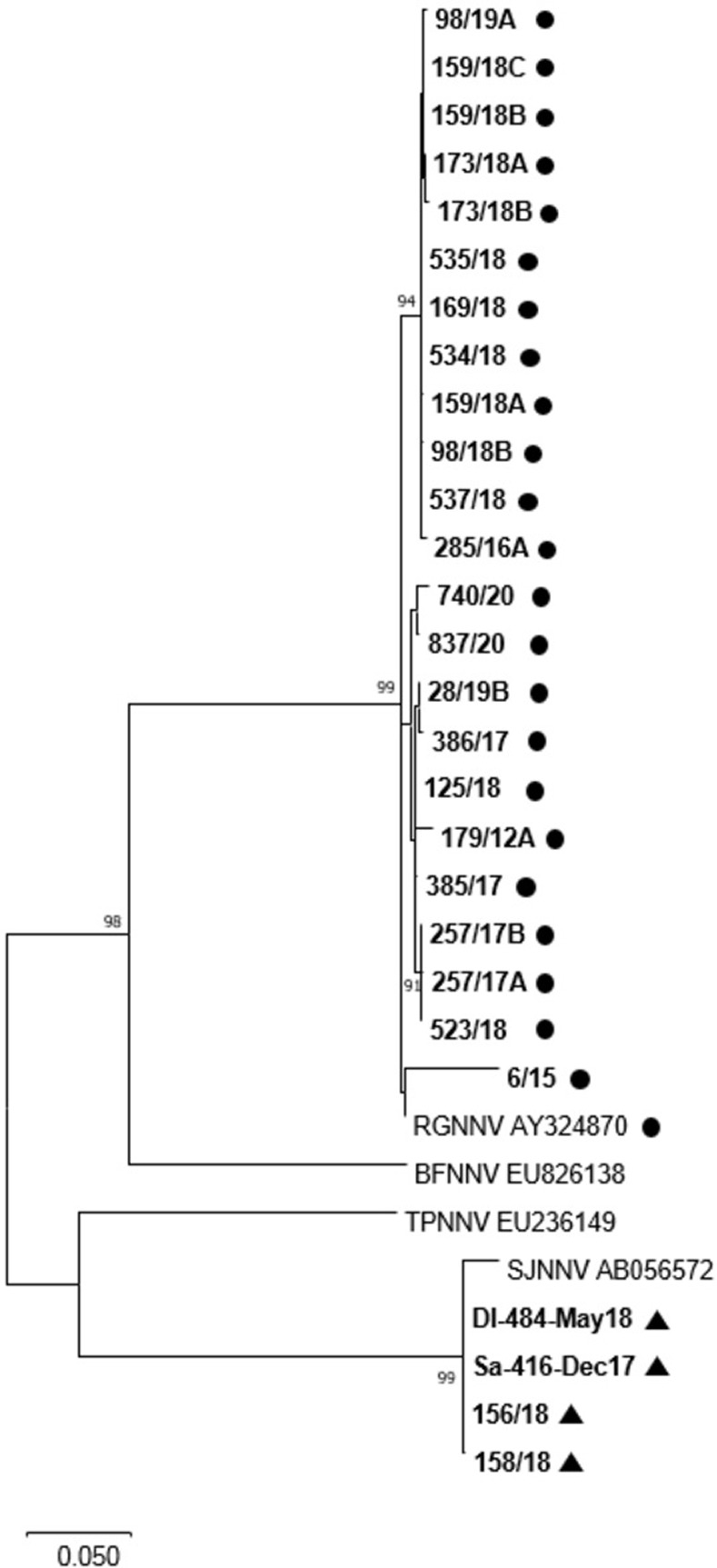
Phylogenetic tree based of RNA2 sequences. Phylogenetic tree based on RNA2 nucleotide sequences. Reference strain are reported with genotype name and accession number. Sequences of tested samples (bold) clustered within the RGNNV or SJNNV group according to their parental RGNNV (dot) or RGNNV/SJNNV reassortant (triangle) nature. Bootstrap values>70% are shown. Branch lengths are scaled according to the number of nucleotide substitutions per site. The scale bar is reported.

Sequencing of an RNA2 fragment from the positive samples confirmed that the strains identified as RGNNV by mRT-PCR had RGNNV-type RNA2, whereas samples identified as reassortants had SJNNV-type RNA2 (Fig 6). Regarding the samples not fully genotyped by the mRT-PCR, all of them were identified as RGNNV genotypes (Fig 6).

Sequence analysis in the primer region targeting the RGNNV-RNA2 of three ungenotyped samples showed 1 nucleotide mismatch at the 3’ end of the forward primer (RG_SPCF1b_RNA2). However, this mismatch was present also in 6 out of 19 sequences of samples correctly genotyped by the mRT-PCR (S1 Fig). For one sequence (sample 179/12) it was possible to obtain only the nested PCR product that does not include the forward primer region.

## Discussion

Nervous necrosis virus is one of the most threatening pathogens of Mediterranean aquaculture. Since its emergence in the late 1980s, the virus has rapidly and broadly evolved, leading to the emergence of new viral variants, including two reassortant strains. Furthermore, the commercial trade of live aquatic animals has significantly contributed to the diffusion of the NNV and its variants together with maritime transport means and migration of the wild hosts [46].

Currently the Mediterranean Basin contains the majority of the viral variances, including the reassortant strains [2].

The increasing variability of NNV strains circulating in aquaculture calls for a continuous upgrade of diagnostic methods. Currently, NNV diagnosis relies on a real-time RT-PCR method validated by the OIE reference laboratory, which provides a quick, sensitive and reliable method to detect NNV being able to amplify and then detect all variants so far described. However, identifying the genotypes and reassortant strains is so far based on conventional genotyping that consists in amplifying and sequencing of both viral genome RNA molecules. Only the analysis of both viral genome segments (RNA1 and RNA2) led to reassortant identification. This method is time-consuming and requires highly specialised staff and equipment, resulting in a limited number of laboratories able to perform a complete and correct viral species identification [19]. For this reason, the availability of a quick, cheap and practical method to distinguish NNV variants, including reassortant strains, could significantly contribute to improve NNV diagnosis capabilities.

In this study, a one-step multiplex RT-PCR targeting RGNNV-RNA1, RGNNV-RNA2 and SJNNV-RNA2 was successfully developed. The method detects the presence and identifies RGNNV genotype and reassortant RGNNV/SJNNV strain and differentiates them from SJNNV genotype and the reassortant SJNNV/RGNNV strain in a single PCR reaction.

The method showed optimal specificity without cross-reaction against viruses and bacteria frequently associated with gilthead seabream, European seabass and marine environment such as LCDV, VHSV, *V*. *anguillarum*, *V*. *harveyi*, *V*. *alginolyticus*, *V*. *gigantis*, *P*. *damselae* subsp. *piscicida* and *A*. *veronii*. Furthermore, the method showed a fairly high sensitivity according to the detection limit reported for other mRT-PCR assays [29,47] and can be applied to confirm VNN field outbreaks.

Moreover, the method showed a high identification capacity. Most of the positive samples (30/34) were detected and fully characterised at RNA1 and RNA2 level. On the other hand, few samples (4/34) were detected and partially characterised being identified only at RNA1 level. The lack of amplification of the RGNNV-RNA2 specific band in these samples could be due to a combination of causes: lower efficiency of the primer pair amplifying this fragment compared to the other primer pairs, long size of the product, presence of 1 mismatch in some sequences and low amount of target RNA in some samples.

The simultaneous detection of several viral targets with a multiplex PCR approach is relatively rapid and cost-effective compared to standard virological and other molecular assays. The mRT-PCR assay developed was carried out in a mix reaction volume equivalent to that of a singleplex; in this way, the cost and the time of execution of the test for the simultaneous detection and identification of NNV and its variants circulating in the Mediterranean are equivalent to those necessary to test only one target leading to a considerable saving of time and costs. Moreover, the developed assay using a so widespread technology as conventional PCR can found a wide implementation in a large number of laboratories with different tecnological levels.

Six finfish species are currently being reared and commercialised in the Mediterranean. Farms in the region commonly operate rearing several species in the same on-growing site, being most of the times gilthead seabream and European seas bass, and most recently meagre (*Argyrosomus regius*), in which has also been detected NNV [23]. Due to the wide range of NNV-susceptible species, the proposed multiplex RT-PCR test could be potentially helpful to understand inter-species transmissions in farming conditions. In fact, the novel assay can be applied to detect and simultaneously genotype viral strains causing an outbreak which involves different species.

Moreover, the proposed method could be applied sequentially with the already OIE validated RT-qPCR method in case of positivity providing NNV genotype information and saving time in comparison to conventional genotyping approach.

The need to discriminate between NNV variants lies in the differences in virulence and pathogenicity of each variant. Furthermore, the developed one-step multiplex RT-PCR is suitable for genotyping a numerous of samples unlike gene sequencing analyses. Therefore, it could support epidemiological studies on NNV variants circulating in the Mediterranean, expanding the knowledge on this virus with reference to its behaviour and distribution. This approach was successfully applied on a large scale survey to investigate the distribution of NNV genotype in Korean shellfish [28].

In conclusion, the developed multiplex RT-PCR represents an easy, rapid, and affordable method to support NNV detection and identification that could find wide application in several sectors of the study of the VNN contributing to improve its knowledge and therefore leading to a better disease control. The method can find wide application for confirmatory diagnosis in NNV outbreaks in the Mediterranean increasing laboratories’ capacity to correctly identify the betanodavirus genotype.

## Supporting information

S1 Fig RNA2 sequence alignment of samples belonging to RGNNV genotype and the primer RG_SPCF1b_RNA2. Primer overlap are shown in bold, mismatches with primer are highlighted in blue. Sequences presenting the mismatch and correctly genotyped by the developed mRT-PCR are in green, whereas sequences presenting the mismatch but ungenotyped by the developed mRT-PCR are in red.
(PDF)

S1 Table RNA1 sequences used for primer design. The Table reports the details of the RNA1 sequences used for primer design: isolate name, genotype, GenBank accession number and the host species.
(DOCX)

S2 Table RNA2 sequences used for primer design. The Table reports the details of the RNA2 sequences used for primer design: isolate name, genotype, GenBank accession number and the host species.
(DOCX)

S3 Table Samples used to validate the multiplex RT-PCR assay. The Table reports the details of the samples used to validate the multiplexRT-PCR assay.
(XLSX)

S4 Table VER-IPT samples tested with the multiplex RT-PCR assay. Details of VER-IPT samples. The table has been modified from Toffan and colleagues [19].
(DOCX)

S1 Raw images (PDF)
