# Supplementary material for: Development and diagnostic validation of a one-step multiplex RT-PCR assay as a rapid method to detect and identify Nervous Necrosis Virus (NNV) and its variants circulating in the Mediterranean
Source: PLoS One. 2022 Aug 26;17(8):e0273802. doi: 10.1371/journal.pone.0273802 (PMC9417010; doi:10.1371/journal.pone.0273802)
Supplement: S1 Fig — Primer overlap are shown in bold, mismatches with primer are highlighted in blue. Sequences presenting the mismatch and correctly genotyped by the developed mRT-PCR are in green, whereas sequences presenting the mismatch but ungenotyped by the developed mRT-PCR are in red. (PDF) [file pone.0273802.s001.pdf]

	....|....|	....|....|	....|....|	....|....|	....|....|
	60	70	80	90	100
6/15	TGCTAACAAT	CGTCGGCGTA	GTAATCGCAC	TGACGCACCC	GTGTTGACTG
98/18B	TGCTAACAAT	CGTCGGCGTA	GTAATCGCAC	TGACGCACCT	GTGTCAAAGG
98/19A	TGCTAACAAT	CGTCGGCGTA	GTAATCGCAC	TGACGCACCT	GTGTCAAAGG
159/18A	TGCTAACAAT	CGTCGGCGTA	GTAATCGCAC	TGACGCACCT	GTGTCAAAGG
159/18B	TGCTAACAAT	CGTCGGCGTA	GTAATCGCAC	TGACGCACCT	GTGTCAAAGG
159/18C	TGCTAACAAT	CGTCGGCGTA	GTAATCGCAC	TGACGCACCT	GTGTCAAAGG
169/18	TGCTAACAAT	CGTCGGCGTA	GTAATCGCAC	TGACGCACCT	GTGTCAAAGG
173/18A	TGCTAACAAT	CGTCGGCGTA	GTAATCGCAC	TGACGCACCT	GTGTCAAAGG
173/18B	TGCTAACAAT	CGTCGGCGTA	GTAATCGCAC	TGACGCACCT	GTGTCAAAGG
285/16A	TGCTAACAAT	CGTCGGCGTA	GTAATCGCAC	TGACGCACCC	GTGTCAAATG
**28/19B**	TGCTAACAAT	CGTCGGCGTA	GTAACCGCAC	TGACGCACCT	GTGTCTAAGG
**125/18**	TGCTAACAAT	CGTCGGCGTA	GTAACCGCAC	TGACGCACCT	GTGTCTAAGG
**523/18**	TGCTAACAAT	CGTCGGCGTA	GTAACCGCAC	TGACGCACCT	GTGTCTAAGG
**257/17A**	TGCTAACAAT	CGTCGGCGTA	GTAACCGCAC	TGACGCACCT	GTGTCTAAGG
**740/20**	TGCTAACAAT	CGTCGGCGTA	GTAACCGCAC	TGACGCACCT	GTGTCTAAGG
**837/20**	TGCTAACAAT	CGTCGGCGTA	GTAACCGCAC	TGACGCACCT	GTGTCTAAGG
**257/17B**	TGCTAACAAT	CGTCGGCGTA	GTAACCGCAC	TGACGCACCT	GTGTCTAAGG
**385/17**	TGCTAACAAT	CGTCGGCGTA	GTAACCGCAC	TGACGCACCT	GTGTCTAAGG
**386/17**	TGCTAACAAT	CGTCGGCGTA	GTAACCGCAC	TGACGCACCT	GTGTCTAAGG
534/18	TGCTAACAAT	CGTCGGCGTA	GTAATCGCAC	TGACGCACCT	GTGTCAAAGG
535/18	TGCTAACAAT	CGTCGGCGTA	GTAATCGCAC	TGACGCACCT	GTGTCAAAGG
537/18	TGCTAACAAT	CGTCGGCGTA	GTAATCGCAC	TGACGCACCT	GTGTCAAAGG
RG_SPCF1b_RNA2	------CAAT	CGTCGGCGTA	GTAATC----	----------	----------
