# Supplementary material for: Development and diagnostic validation of a one-step multiplex RT-PCR assay as a rapid method to detect and identify Nervous Necrosis Virus (NNV) and its variants circulating in the Mediterranean
Source: PLoS One. 2022 Aug 26;17(8):e0273802. doi: 10.1371/journal.pone.0273802 (PMC9417010; doi:10.1371/journal.pone.0273802)
Supplement: S1 Table — The Table reports the details of the RNA1 sequences used for primer design: isolate name, genotype, GenBank accession number and the host species. (DOCX) [file pone.0273802.s002.docx]

**Table S1. RNA1 sequences used for primer design**

| **Isolate** | **Genotype** | **Accession number** | **Host species** |
| --- | --- | --- | --- |
| 389/l96 | SJNNV/RGNNV | KF386163.1 | *Dicentrarchus labrax* |
| 484.2.2009 | SJNNV/SJNNV | JN189814.2 | *Solea senegalensis* |
| Striped Jack nervous necrosis virus | SJNNV/SJNNV | AB025018.1 | *Pseudocaranx dentex* |
| Striped Jack nervous necrosis virus | SJNNV/SJNNV | AB056571.1 | *Pseudocaranx dentex* |
| Striped Jack nervous necrosis virus | SJNNV/SJNNV | NC_003448.1 | *Pseudocaranx dentex* |
| 6.1.2007 | RGNNV/RGNNV | JN189857.1 | *Dicentrarchus labrax* |
| 31.1.2007 | RGNNV/RGNNV | JN189799.1 | *Mullus barbatus* |
| 100.1.2003 | RGNNV/RGNNV | JN189858.1 | *Dicentrarchus labrax* |
| 312.1.2005 | RGNNV/RGNNV | JN189869.1 | *Mullus* sp. |
| 424.1.2003 | RGNNV/RGNNV | JN189883.1 | *Balistapus* sp. |
| 628.1.2005 | RGNNV/RGNNV | JN189893.1 | *Salmo trutta trutta* |
| 332.2.2006 | RGNNV/RGNNV | JN189872.1 | *Dicentrarchus labrax* |
| 412.2.2005 | RGNNV/RGNNV | JN189881.1 | *Mullus* sp. |
| 550.2.2005 | RGNNV/RGNNV | JN189823.1 | *Epinephelus* sp. |
| 316.3.2007 | RGNNV/RGNNV | JN189817.1 | *Dicentrarchus labrax* |
| 373.1.3.2004 | RGNNV/RGNNV | JN189877.1 | *Mullus barbatus* |
| 390.3.2003 | RGNNV/RGNNV | JN189910.1 | *Solea solea* |
| 45.5.2005 | RGNNV/RGNNV | JN189887.1 | *Gobius* sp. |
| 334.6.2009 | RGNNV/RGNNV | JN189841.1 | *Dicentrarchus labrax* |
| 505.6.2004 | RGNNV/RGNNV | JN189891.1 | *Mullus barbatus* |
| 8.2005 | RGNNV/RGNNV | JN189895.1 | *Pagellus* sp. |
| 39.13.2009 | RGNNV/RGNNV | JN189803.1 | *Dicentrarchus labrax* |
| 285.13.2009 | RGNNV/RGNNV | JN189866.1 | *Ruditapes philippinarum* |
| 474.23.2008 | RGNNV/RGNNV | JN189808.1 | *Mullus barbatus* |
| 283.2009 | RGNNV/RGNNV | JN189865.2 | *Dicentrarchus labrax* |
| 289.2002 | RGNNV/RGNNV | JN189905.1 | *Umbrina cirrosa* |
| 384.2007 | RGNNV/RGNNV | JN189822.1 | *Dicentrarchus labrax* |
| 410.2006 | RGNNV/RGNNV | JN189853.1 | *Dicentrarchus labrax* |
| 512.2000 | RGNNV/RGNNV | JN189892.1 | *Dicentrarchus labrax* |
| Dl-1 | RGNNV/RGNNV | AM085326.1 | *Dicentrarchus labrax* |
| Dl-l-00b | RGNNV/RGNNV | AM085325.1 | *Dicentrarchus labrax* |
| Dl-I-96a | RGNNV/RGNNV | AM085327.1 | *Dicentrarchus labrax* |
| Sa-l-00 | RGNNV/RGNNV | AM085314.1 | *Sparus aurata* |
| SpDl Iausc168808 | RGNNV/RGNNV | FJ803915.1 | *Dicentrarchus labrax* |
| SGWak97 | RGNNV/RGNNV | AY324869.1 | *Hyporthodus septemfasciatus* |
| Uc-1 | RGNNV/RGNNV | AM085313.1 | *Umbrina cirrosa* |
| 17.1C.2004 | RGNNV/SJNNV | JN189900.1 | *Dicentrarchus labrax* |
| 24.1.2005 | RGNNV/SJNNV | JN189844.1 | *Sparus aurata* |
| 367.2.2005 | RGNNV/SJNNV | JN189909.2 | *Dicentrarchus labrax* |
| 69.3.2009 | RGNNV/SJNNV | JN189914.1 | *Opistobranchia* |
| 250.3.2009 | RGNNV/SJNNV | JN189830.1 | *Artemia salina* |
| 82.4.2007 | RGNNV/SJNNV | JX290516.1 | *Sparus aurata* |
| 292.7.8.2009 | RGNNV/SJNNV | JN189833.1 | *Dicentrarchus labrax* |
| 28.2005 | RGNNV/SJNNV | JN189904.1 | *Solea senegalensis* |
| 132.2005 | RGNNV/SJNNV | JN189899.1 | *Dicentrarchus labrax* |
| 430.2004 | RGNNV/SJNNV | JN189911.1 | *Solea senegalensis* |
| 446.2005 | RGNNV/SJNNV | JN189885.1 | *Solea solea* |
| 477.2004 | RGNNV/SJNNV | JN189913.1 | *Solea solea* |
| PtSa Iausc6105 | RGNNV/SJNNV | FJ803912.1 | *Sparus aurata* |
| PtSs Iausc57304 | RGNNV/SJNNV | FJ803914.1 | *Solea senegalensis* |
| SpSs Iausc197408 | RGNNV/SJNNV | FJ803917.1 | *Solea senegalensis* |
| VNNV/S.aurata/CY/203-3/May2010 | RGNNV/SJNNV | KY354685.1 | *Sparus aurata* |
| VNNV/S.aurata/Farm1/127-1/Mar2015 | RGNNV/SJNNV | KY354691.1 | *Sparus aurata* |
| VNNV/S.aurata/Farm1/461-1/Nov2014 | RGNNV/SJNNV | KY354688.1 | *Sparus aurata* |
| VNNV/S.aurata/Farm2/165-6/Mar2016 | RGNNV/SJNNV | KY354693.1 | *Sparus aurata* |
| VNNV/S.aurata/Farm2/575/Nov2015 | RGNNV/SJNNV | KY354692.1 | *Sparus aurata* |
| VNNV/S.aurata/I/69-4/Mar2009 | RGNNV/SJNNV | KY354683.1 | *Sparus aurata* |
