# Supplementary material for: Development and diagnostic validation of a one-step multiplex RT-PCR assay as a rapid method to detect and identify Nervous Necrosis Virus (NNV) and its variants circulating in the Mediterranean
Source: PLoS One. 2022 Aug 26;17(8):e0273802. doi: 10.1371/journal.pone.0273802 (PMC9417010; doi:10.1371/journal.pone.0273802)
Supplement: S2 Table — The Table reports the details of the RNA2 sequences used for primer design: isolate name, genotype, GenBank accession number and the host species. (DOCX) [file pone.0273802.s003.docx]

| **Isolate** | **Genotype** | **Accession number** | **Host species** |
| --- | --- | --- | --- |
| 389/l96 | SJNNV/RGNNV | KF386164.1 | *Dicentrarchus labrax* |
| 484.2.2009 | SJNNV/SJNNV | JN189919.2 | *Solea senegalensis* |
| JP06 | SJNNV/SJNNV | AF175519.1 | *Dicentrarchus labrax* |
| Striped Jack nervous necrosis virus | SJNNV/SJNNV | AB056572.1 | *Pseudocaranx dentex* |
| Striped Jack nervous necrosis virus | SJNNV/SJNNV | NC_003449.1 | *Pseudocaranx dentex* |
| 6.1.2007 | RGNNV/RGNNV | JN189946.1 | *Dicentrarchus labrax* |
| 8.2005 | RGNNV/RGNNV | JN190017.1 | *Pagellus* sp. |
| 31.1.2007 | RGNNV/RGNNV | JN189952.1 | *Mullus barbatus* |
| 100.1.2003 | RGNNV/RGNNV | JN190018.1 | *Dicentrarchus labrax* |
| 283.2009 | RGNNV/RGNNV | JN189992.2 | *Dicentrarchus labrax* |
| 285.13.2009 | RGNNV/RGNNV | JN189993.1 | *Ruditapes philippinarum* |
| 289.2002 | RGNNV/RGNNV | JN190008.1 | *Umbrina cirrosa* |
| 312.1.2005 | RGNNV/RGNNV | JN189995.1 | *Mullus* sp. |
| 316.3.2007 | RGNNV/RGNNV | JN189969.1 | *Dicentrarchus labrax* |
| 332.2.2006 | RGNNV/RGNNV | JN190012.1 | *Dicentrarchus labrax* |
| 334.6.2009 | RGNNV/RGNNV | JN189988.1 | *Dicentrarchus labrax* |
| 384.2007 | RGNNV/RGNNV | JN189974.1 | *Dicentrarchus labrax* |
| 39.13.2009 | RGNNV/RGNNV | JN189956.1 | *Dicentrarchus labrax* |
| 390.3.2003 | RGNNV/RGNNV | JN190024.1 | *Solea solea* |
| 410.2006 | RGNNV/RGNNV | JN189943.1 | *Dicentrarchus labrax* |
| 412.2.2005 | RGNNV/RGNNV | JN190026.1 | *Mullus* sp. |
| 424.1.2003 | RGNNV/RGNNV | JN190014.1 | *Balistapus* sp. |
| 45.5.2005 | RGNNV/RGNNV | JN190002.1 | *Gobius* sp. |
| 474.23.2008 | RGNNV/RGNNV | JN189962.1 | *Mullus barbatus* |
| 505.6.2004 | RGNNV/RGNNV | JN190003.1 | *Mullus barbatus* |
| 512.2000 | RGNNV/RGNNV | JN190031.1 | *Dicentrarchus labrax* |
| 550.2.2005 | RGNNV/RGNNV | JN189975.1 | *Epinephelus* sp. |
| 628.1.2005 | RGNNV/RGNNV | JN190032.1 | *Salmo trutta trutta* |
| Dl-1 | RGNNV/RGNNV | AJ277803.1 | *Dicentrarchus labrax* |
| Dl-l-96a | RGNNV/RGNNV | AM085342.1 | *Dicentrarchus labrax* |
| It/351/Sb | RGNNV/RGNNV | AY620367.1 | *Dicentrarchus labrax* |
| RG-TO91 | RGNNV/RGNNV | D38636.1 | *Epinephelus akaara* |
| Sa-l-00 | RGNNV/RGNNV | AM085338.1 | *Sparus aurata* |
| SGWak97 | RGNNV/RGNNV | AY324870.1 | *Hyporthodus septemfasciatus* |
| SpDl_Iausc168808 | RGNNV/RGNNV | FJ829452.1 | *Dicentrarchus labrax* |
| Uc-1 | RGNNV/RGNNV | AJ277811.1 | *Umbrina cirrosa* |
| 17.1C.2004 | RGNNV/SJNNV | JN189934.1 | *Dicentrarchus labrax* |
| 24.1.2005 | RGNNV/SJNNV | JN189916.1 | *Sparus aurata* |
| 28.2005 | RGNNV/SJNNV | JN189935.1 | *Solea senegalensis* |
| 132.2005 | RGNNV/SJNNV | JN189937.1 | *Dicentrarchus labrax* |
| 367.2.2005 | RGNNV/SJNNV | JN189936.2 | *Dicentrarchus labrax* |
| 430.2004 | RGNNV/SJNNV | JN189932.1 | *Solea senegalensis* |
| 446.2005 | RGNNV/SJNNV | JN189930.1 | *Solea solea* |
| 477.2004 | RGNNV/SJNNV | JN189938.1 | *Solea solea* |
| Ptsa_Iausc6105 | RGNNV/SJNNV | FJ803918.1 | *Sparus aurata* |
| PtSs_Iausc57304 | RGNNV/SJNNV | FJ803920.1 | *Solea senegalensis* |
| Sa-416-Dec17 | RGNNV/SJNNV | MN896009.1 | *Sparus auratA* |
| SpSs_Iausc197408 | RGNNV/SJNNV | FJ803922.1 | *Solea senegalensis* |
| VNNV/S.aurata/l/69-4/Mar2009 | RGNNV/SJNNV | KY354695.1 | *Sparus aurata* |

**Table S2. RNA2 sequences used for primer design**
