# Supplementary material for: Development and diagnostic validation of a one-step multiplex RT-PCR assay as a rapid method to detect and identify Nervous Necrosis Virus (NNV) and its variants circulating in the Mediterranean
Source: PLoS One. 2022 Aug 26;17(8):e0273802. doi: 10.1371/journal.pone.0273802 (PMC9417010; doi:10.1371/journal.pone.0273802)
Supplement: S4 Table — Details of VER-IPT samples. The table has been modified from Toffan and colleagues [19]. (DOCX) [file pone.0273802.s005.docx]

| **Sample number** | **Strain/content** | **Genotype** | **Viral titre (TCID_50_ ml^-1^)** |
| --- | --- | --- | --- |
| 1 | 283.2009 | RGNNV | 10^7.30^ |
| 2 | 367.2.2005 | RGNNV/SJNNV | 10^7.55^ |
| 3 | 484.2.2009 | SJNNV | 10^9.55^ |
| 4 | 389/I96 | SJNNV/RGNNV | 10^8.30^ |
| 5 | Sterile MEM+ 10% yeast extract | - | - |
| 6 | 367.2.2005 | RGNNV/SJNNV | 10^4.55^ |
| 7 | Sterile MEM | - | - |
| 8 | Sterile MEM | - | - |
| 9 | Sterile MEM+ 10% yeast extract | - | - |
| 10 | Negative rainbow trout serum | - | - |

**S4 Table.** Details of VER-IPT samples, the table has been modified from Toffan and colleagues [18].
