# Supplementary material for: Development and diagnostic validation of a one-step multiplex RT-PCR assay as a rapid method to detect and identify Nervous Necrosis Virus (NNV) and its variants circulating in the Mediterranean
Source: PLoS One. 2022 Aug 26;17(8):e0273802. doi: 10.1371/journal.pone.0273802 (PMC9417010; doi:10.1371/journal.pone.0273802)
Supplement: S1 Raw images — (PDF) [file pone.0273802.s006.pdf]

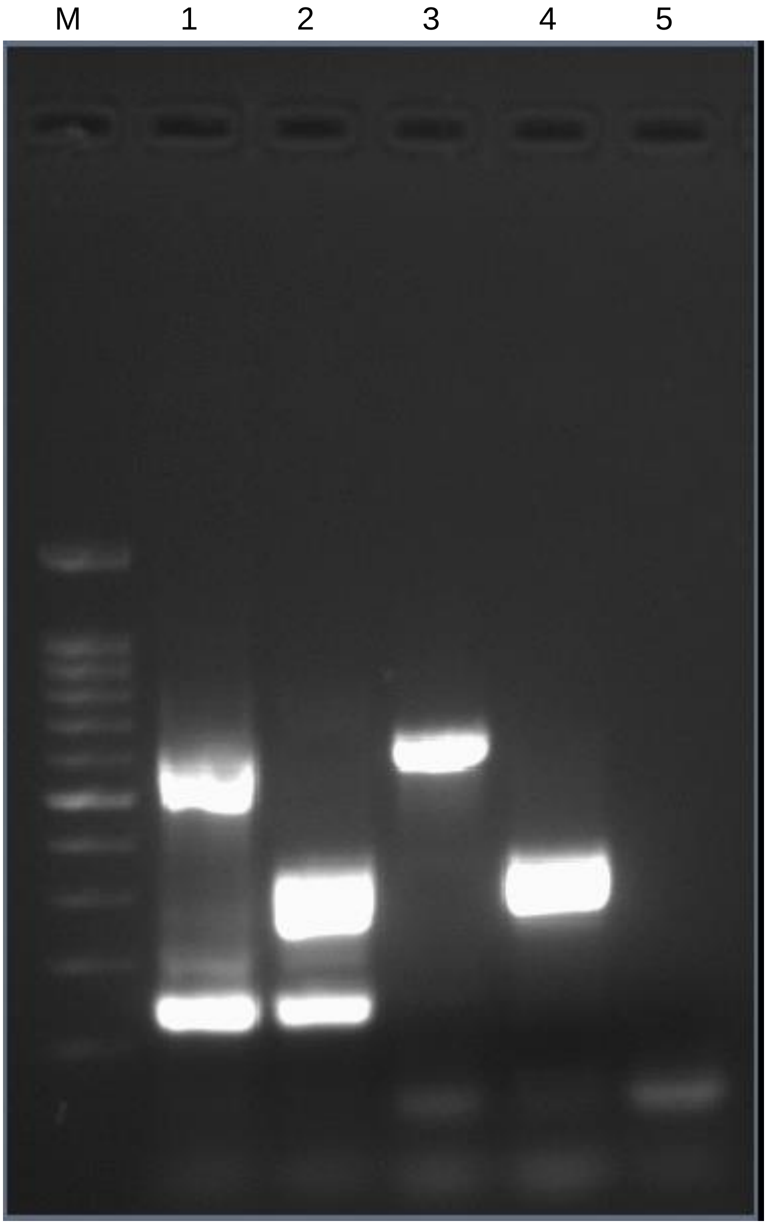

Fig 1 original image. Multiplex RT-PCR. Agarose gel electrophoresis of PCR products amplified from RNA extracted from NNV reference strains. Lane M: 100 bp ladder (Invitrogen, Carlsbad, USA); Lane 1: RGNNV genotype; Lane 2: RGNNV/SJNNV reassortant strain; Lane 3: SJNNV/RGNNV reassortant strain; Lane 4: SJNNV genotype; Lane 5: no template control; Molecular weight markers (bp) are indicated to the left of the gel.

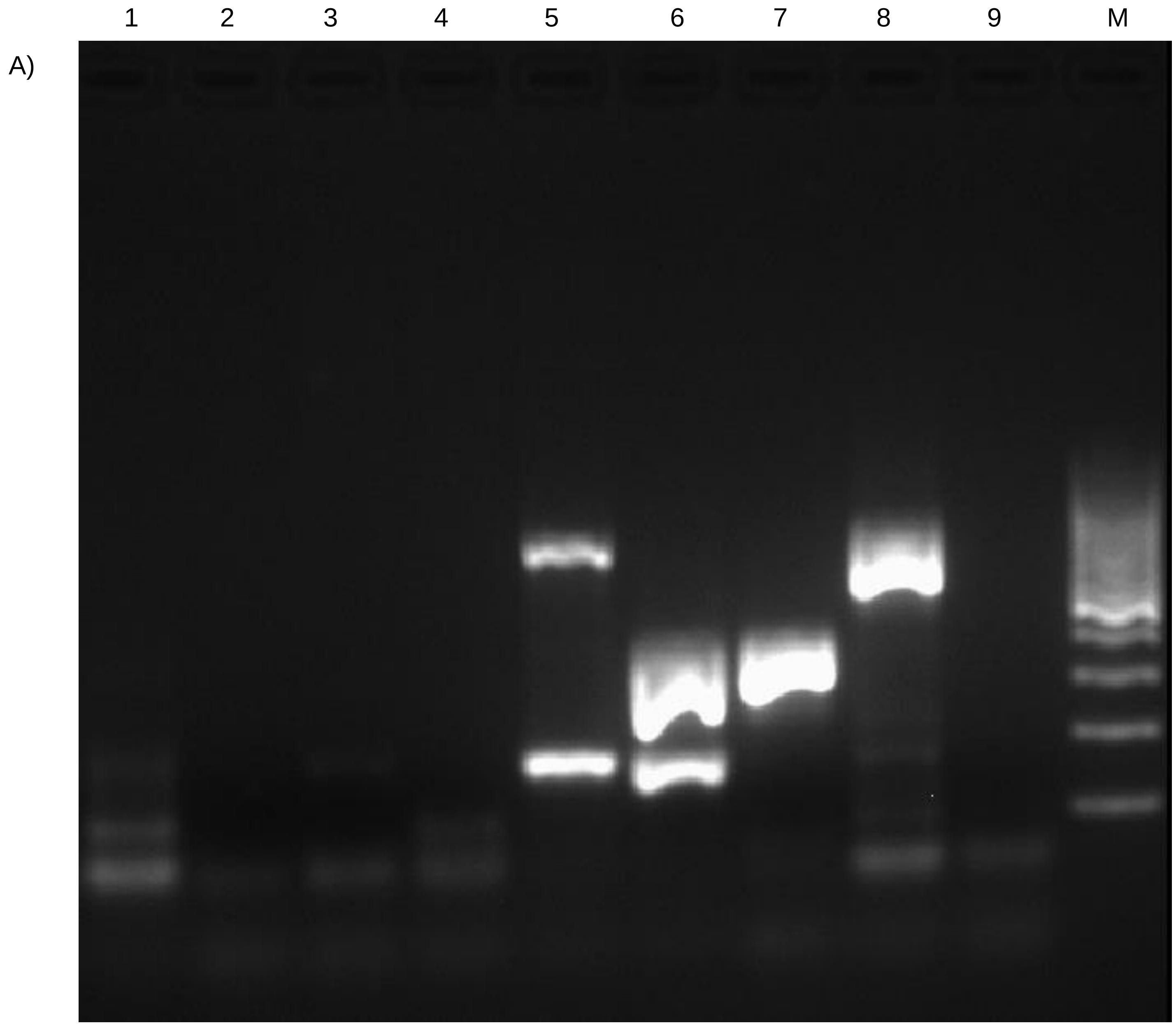

Fig 2. original image. Multiplex RT-PCR specificity test. Agarose gel electrophoresis of PCR products amplified from cell-culture samples and viral and bacterial fish pathogens. A) Lane 1: VHSV; Lane 2: V. *harveyi*; Lane 3: *V. alginolyticus*; Lane 4: *V. gigantis*; Lane 5: RGNNV genotype; Lane 6: RGNNV/SJNNV reassortant strain; Lane 7: SJNNV/RGNNV reassortant strain; Lane 8: SJNNV genotype; Lane 9: no template control.

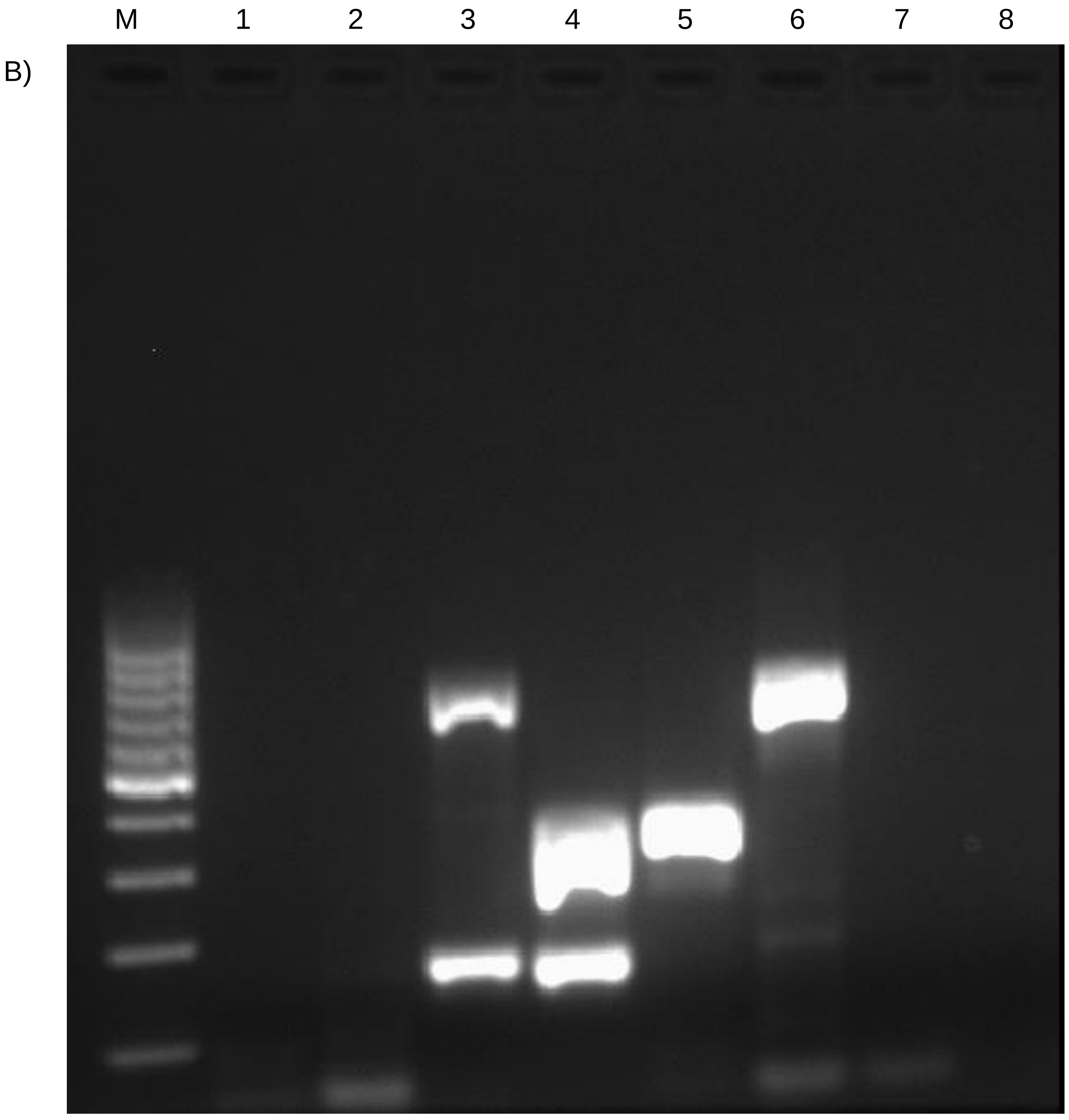

Fig 2. original image Multiplex RT-PCR specificity test. Agarose gel electrophoresis of PCR products amplified from cell-culture samples and viral and bacterial fish pathogens. B) Lane 1: BFNNV; Lane 2: TPNNV; Lane 3: RGNNV genotype; Lane 4: RGNNV/SJNNV reassortant strain; Lane 5: SJNNV/RGNNV reassortant strain; Lane 6: SJNNV genotype; Lane 7: no template control.

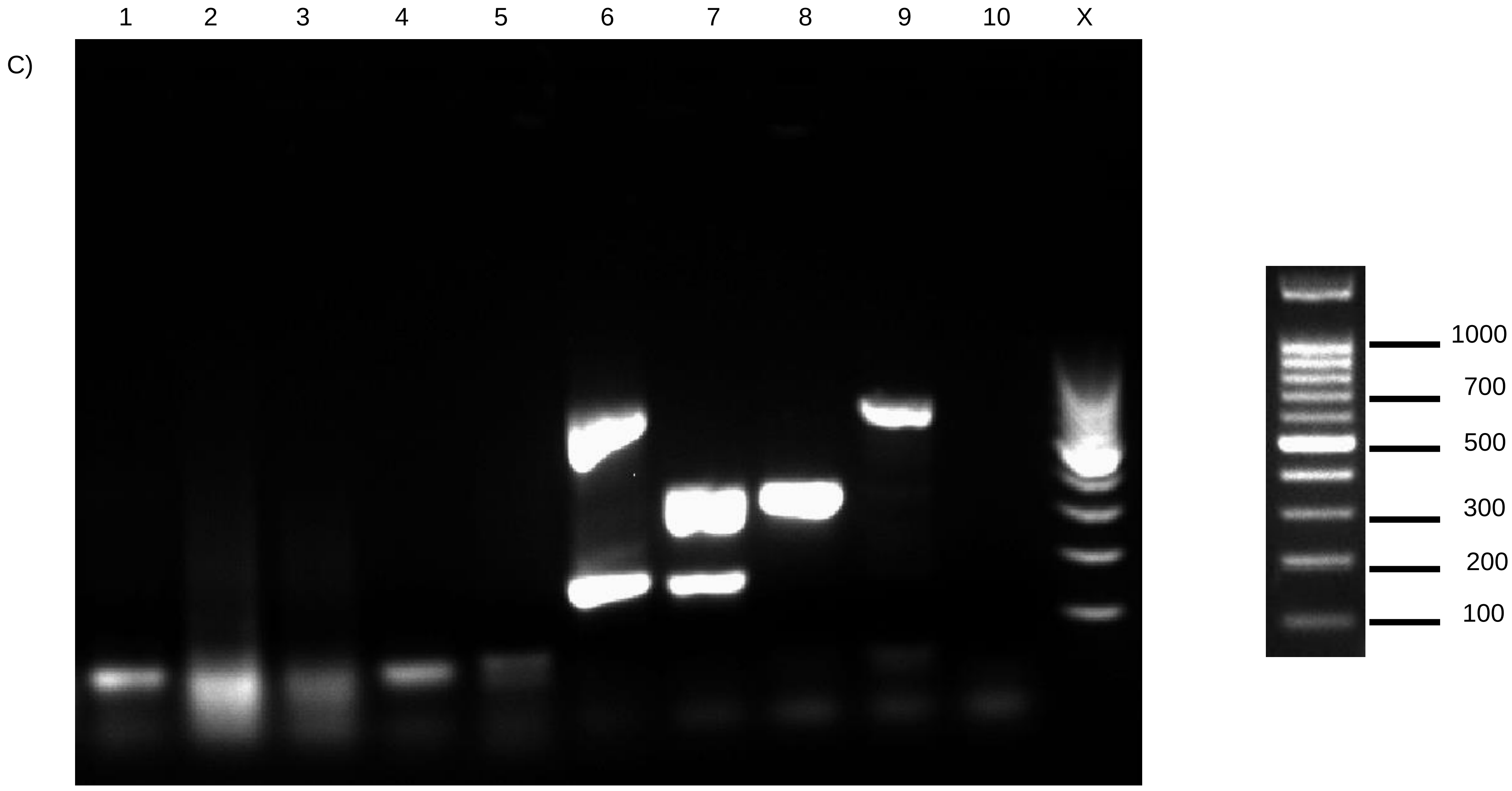

Fig 2. original image. Multiplex RT-PCR specificity test. Agarose gel electrophoresis of PCR products amplified from cell-culture samples and viral and bacterial fish pathogens. C) Lane 1: SSN1 cell line; Lane 2: LCDV; Lane 3: *V. anguillarum*; Lane 4: *P. damselae* subsp. *piscicida*; Lane 5: *A. veronii*; Lane 6: RGNNV genotype; Lane 7: RGNNV/SJNNV reassortant strain; Lane 8: SJNNV/RGNNV reassortant strain; Lane 9: SJNNV genotype; Lane 10: no template control. Lanes M: 100 bp ladder (Invitrogen, Carlsbad, USA).

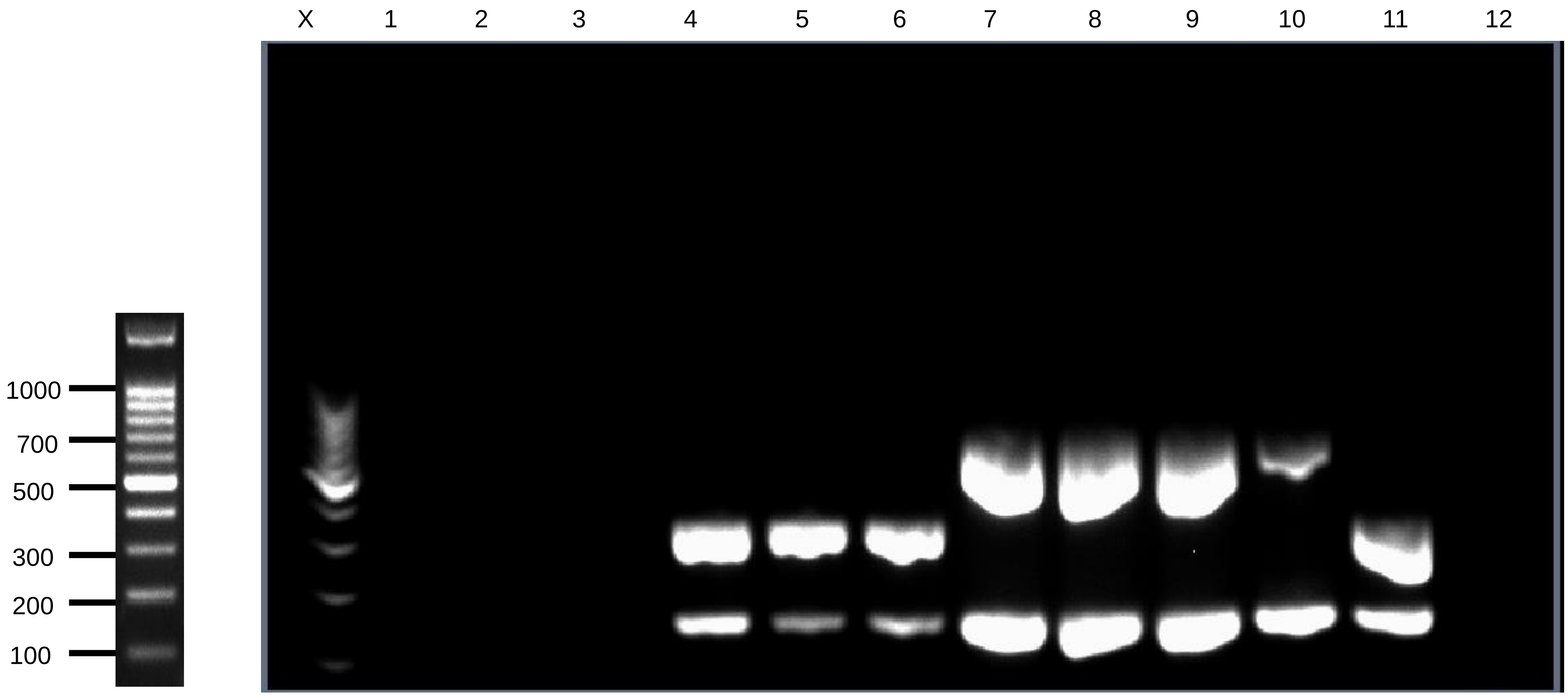

Fig 3. original image. Multiplex RT-PCR reapetibility test. Agarose gel electrophoresis of PCR products amplified from RNA extracted from NNV-positive and NNV-negative samples. Lane M: 100 bp ladder (Invitrogen, Carlsbad, USA); Lane 1-3: NNV-negative sample Lane 4-6: NNV weakly positive sample; Lane 7-9: NNV distinct positive sample; Lane 10: RGNNV genotype; Lane 11: RGNNV/SJNNV reassortant strain; Lane 12: no template control; Molecular weight markers (bp) are indicated to the left of the gel.

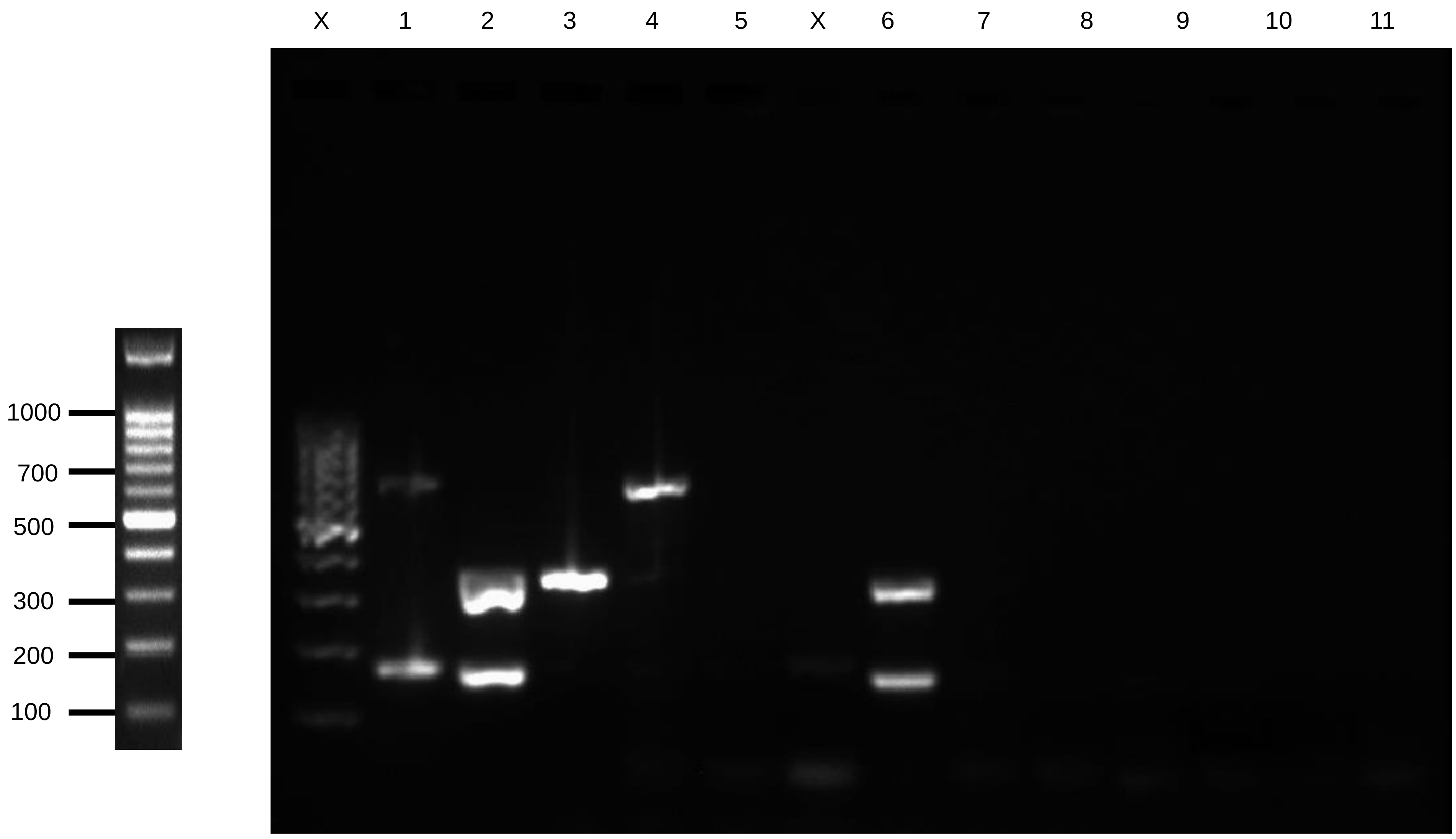

Fig 4. original image. Multiplex RT-PCR applied to VER-IPT samples. Agarose gel electrophoresis of PCR products amplified from RNA extracted from ten VER-IPT samples. Lane M: 100 bp ladder (Invitrogen, Carlsbad, USA); Lane 1-4 and 6: NNV-positive samples Lane 5 and 7-10: NNV-negative samples; Lane 11: no template control; Molecular weight markers (bp) are indicated to the left of the gel. For sample details see S4 Table.
